# Is conduction system pacing improving cardiac performance in patients with right bundle branch block and heart failure?

**DOI:** 10.3389/fphys.2025.1690243

**Published:** 2025-11-25

**Authors:** Jia Jing-Jing, Sun Xi-Xia, Li Tian-Zhu, Wang Xin, Yang Xiao-Lei, Ai Xin-Jing, Xia Yun-Long, Dong Ying-Xue

**Affiliations:** Department of Cardiology, The First Affiliated Hospital of Dalian Medical University, Dalian, China

**Keywords:** conduction system pacing, His-bundle pacing, left bundle branch pacing, cardiac resynchronization therapy, right bundle branch block, heart failure

## Abstract

**Objective:**

The aim of this study was to evaluate the feasibility and safety of conduction system pacing (CSP) in patients with right bundle branch block (RBBB) and heart failure (HF).

**Methods:**

This retrospective study included all the patients with HF and a ventricular pacing frequency of more than 40% who underwent CSP between 2018 and 2023, with all enrolled patients presenting RBBB prior to the procedure. Clinical data, including echocardiographic and electrocardiographic findings, were collected before and after the procedure, with a minimum follow-up duration of 2 years.

**Results:**

CSP was successfully performed in 78 patients (78/88, 88.63%), comprising 13 patients (13/78, 16.67%) with His-bundle pacing (HBP) and 65 patients (65/78, 83.33%) with left bundle branch pacing (LBBP). Significant improvements were observed in QRS duration (148.06 ± 17.91 ms vs. 121.87 ± 14.47 ms, *p* < 0.001), left ventricular ejection fraction (LVEF) (43.79% ± 11.71% vs. 46.94% ± 10.06%, *p* = 0.020), left ventricular end-diastolic diameter (LVEDD) (54.15 ± 7.67 mm vs. 52.71 ± 7.67 mm, = 0.016), and the New York Heart Association (NYHA) functional class (2.97 ± 0.68 vs. 1.63 ± 1.08, *p* = 0.001). No significant change was noted in the left atrial diameter (LAD) (44.72 ± 8.07 mm vs. 43.86 ± 8.42 mm, *p* = 0.114). Subgroup analysis revealed that a marked LVEF improvement was observed in patients with baseline LVEF ≤35% (30.05% ± 2.76% vs. 41.42% ± 11.61%, *p* = 0.001). Logistic regression analysis revealed that LVEF (OR = 0.112, 95% CI: 0.011–0.839, and *p* = 0.001) and ΔQRS (OR = 1.449, 95% CI: 1.292–2.445, and *p* = 0.021) were independent predictors of echocardiographic response.

**Conclusion:**

CSP is safe and effective for patients with RBBB and HF, with particularly notable improvements in cardiac performance among those with severely reduced LVEF.

## Background

It is well established that left bundle branch block (LBBB) induces electrical asynchrony of the left ventricular lateral wall and contributes to the development of heart failure (HF) (Baldasseroni et al., 2002). In contrast, the impact of right bundle branch block (RBBB) on inter- and intraventricular asynchrony remains unclear (Zhu et al., 2022). RBBB arises from structural abnormalities such as sclerosis, fibrosis, or necrosis within the right bundle branch and has been associated with increased all-cause mortality, cardiovascular mortality, and hospitalization for HF (Reddy et al., 2025). Nevertheless, a recent study showed that cardiac resynchronization therapy (CRT) via biventricular pacing (BiVP) provided little to no benefit for patients with HF and RBBB (Biton et al., 2016). These findings highlight the need for personalized evaluation that integrates QRS morphology, HF severity, and indices of asynchrony.

Over the past decade, conduction system pacing (CSP), including His-bundle pacing (HBP) and left bundle branch pacing (LBBP), has emerged as an alternative for patients requiring cardiac electrical resynchronization, particularly those with LBBB and HF (Huang et al., 2017; Vijayaraman et al., 2023). Notably, recent studies have shown that HBP can effectively improve clinical outcomes in patients with RBBB and heart failure with reduced left ventricular ejection fraction (HFrEF) (Sharma et al., 2018a; Sharma et al., 2018b). Similarly, LBBP has also been demonstrated to be associated with improved cardiac performance in patients with RBBB and HF (Vijayaraman et al., 2022). It is important to note that the QRS morphology induced by LBBP often differs from that of intrinsic RBBB, prompting the question of whether LBBP can modulate electrical synchronization in these patients, a possibility that remains unproven. More importantly, data on the efficacy of CSP in patients with RBBB and HF remain limited. Therefore, the present study aims to investigate the feasibility of CSP for improving cardiac performance in patients with HF and RBBB, along with its potential mechanisms.

## Methods

### Patient enrollment

This observational cohort study retrospectively recruited patients with complete RBBB, with a QRS duration exceeding 120 ms, who underwent CSP from May 2018 to May 2023. The exclusion criteria included patients with left bundle branch block, including left anterior fascicular block or left posterior fascicular block, a right ventricular pacing percentage of less than 40%, device upgrades, generator replacements, recent myocardial infarction, and severe valvular disease (including severe mitral or aortic regurgitation/stenosis). This study protocol was approved by the Institutional Ethics Board of our hospital (PJ-KS-KY-2023-181).

#### Procedure and protocol

HBP and LBBP were performed using the SelectSecure Pacing Lead (Model 3830, Medtronic Inc.) and a fixed-curve sheath (C315 HIS, Medtronic Inc.). His bundle and left bundle branch electrograms were recorded in a unipolar configuration (Prucka CardioLab, GE Healthcare), if available. The unipolar paced QRS configuration and pacing impedance were monitored, with concurrent measurement of peak left ventricle activation times (LVATs) in lead V5 for LBBP (Jiang et al., 2020). If CSP failed, according to CRT indications, the left ventricular (LV) lead was placed using a standard technique in the lateral or posterolateral region of the LV. Atrioventricular (AV) delay was programmed, and pacing output voltage was adjusted to achieve the shortest QRS duration and satisfy QRS morphology.

### Criteria and definition

An abrupt reduction in LVAT of more than 10 ms, with QRS morphologies of qR or rSR’ in lead V_1_, was the simple criterion for LBBP (Jiang et al., 2020). Criteria for HBP are defined as a paced QRS morphology concordant with the intrinsic QRS or as complete reversal of RBBB (Zhang et al., 2018). Left axis deviation was defined as the QRS axis of −30 to −90°, while right axis deviation was 90–180° (Perloff and Cabeen, 1979). RBBB was defined as a QRS duration of ≥120 ms, with a deep terminal S wave in leads I and V_6_ and an RSR’, wide R, or qR pattern in lead V_1_ (Rickard et al., 2011). Heart failure is defined as a clinical syndrome characterized by cardinal symptoms and signs, and heart failure with preserved ejection fraction (HFpEF) and those with LVEF < 50% were all included (McDonagh et al., 2021). Echocardiographic response was defined as a ≥5% increase in LVEF (Ellenbogen and Huizar, 2012).

Complete reversal of RBBB is defined as normal QRS morphology (including normal axis, appropriate transition, and duration ≤110 ms) with an rS morphology in lead V_1_ (Vijayaraman et al., 2019; Huang et al., 2019). Incomplete reversal of RBBB is defined as modulated paced RBBB morphology, featuring a shorter QRS duration in lead V_1_ and a shorter S duration in leads V_6_ and I (Wu et al., 2021). Pacing threshold increment was defined as the minimal absolute increase in 1V @0.4 ms during follow-up.

## Data collection and follow-up

Baseline demographics and medical histories were collected at enrollment, including patient characteristics and procedure success rates. During the operation and follow-up, pacemaker parameters were recorded. Device follow-up monitored electrocardiographic parameters and lead-related complications (lead dislodgement, loss of capture, and significant increases in pacing threshold). Throughout follow-up, 12-lead electrocardiograms (ECGs), complications, and pacemaker parameters were continuously monitored. Echocardiography was scheduled at 6, 12, and 24 months after the procedure. Adverse events (thrombosis, infection, lead dislodgement, perforation, stroke, and death) were documented, with a minimum follow-up duration of 2 years.

### Statistical analysis

- Continuous variables are expressed as the mean ± standard deviation (SD) and were compared using paired *t*-tests for normally distributed data. Categorical variables are expressed as percentages (%) and were compared using χ^2^ tests. Nonparametric tests were used for non-normally distributed data. One-way analysis of variance is used to compare changes in relevant indicators across different time points. Univariate and multivariable logistic regression analyses were used to identify predictors of echocardiographic response, with univariate predictors showing a *p*-value less than 0.05 incorporated into the multivariate logistic regression analysis. All statistical analyses were performed using SPSS version 26.0, with a significance threshold set at *p* < 0.05.

## Results

A total of 88 patients were enrolled, among whom 10 patients failed in RBBB correction.

### Baseline characteristics

Baseline characteristics of the study population are presented in Table 1. The mean age of patients was 72.59 ± 9.51 years, with 67.90% being male. A total of 48 patients (61.54%) had LVEF< 50%, and 14 patients (17.95%) had ischemic cardiomyopathy. The baseline QRS duration was 148.06 ± 17.91 ms. Of the 88 patients, 78 (88.6%) underwent successful CSP. The average follow-up duration was 26.94 ± 9.12 months. During the follow-up period, 5 patients (6.4%) experienced all-cause mortality, and 10 patients (12.82%) were readmitted to the hospital due to heart failure.

**TABLE 1:** Baseline characteristics of enrolled patients.

Characteristic	
Male	53 (67.90%)
Age (y)	72.59 ± 9.51
BMI (Kg/m^2^)	24.01 ± 4.88
Hypertension	44 (56.41%)
Diabetes mellitus	18 (23.07%)
Atrial fibrillation	43 (55.13%)
Ischemic cardiomyopathy	14 (17.95%)
Dilated cardiomyopathy	8 (10.26%)
CR (umol/L)	88.97 ± 34.37
BNP (ng/L)	3,542.71 ± 2,269.22
NYHAⅡ, n (%)	17 (21.79)
NYHA Ⅲ, n (%)	45 (57.7)
NYHA IV, n (%)	16 (20.5)
LVEF ≤ 35%	22 (32.35)
LVEF < 50%, n (%)	48 (61.54)
LVEF ≥ 50%, n (%)	30 (38.46)
QRSd (ms)	148.06 ± 17.91
Follow-up duration (months)	26.94 ± 9.12

BMI, body mass index; CR, creatinine; BNP, B-type natriuretic peptide; LVEF, left ventricular ejection fraction; NYHA, New York Heart Association; QRSd, QRS duration.

The use of angiotensin-converting enzyme inhibitors (ACEIs)/angiotensin receptor blockers (ARBs)/angiotensin receptor–neprilysin inhibitors (ARNIs) (64.10% vs. 66.67%, *p* = 0.866), mineralocorticoid receptor antagonists (MRAs) (48.71% vs. 43.58%, *p* = 0.630), and sodium–glucose cotransporter 2 inhibitor (SGLT2i) (82.05% vs. 78.20%, *p* = 0.689) was not significantly different; however, the use of beta-blockers was 42.30% during follow-up. All patients received guideline-directed medical therapy adjusted for blood pressure, heart rate, renal function, electrolytes, and comorbidities, following the heart failure guidelines. The medications were titrated to the maximum tolerated dose for each patient according to their specific circumstances (McDonagh et al., 2021).

Compared with those in whom RBBB correction was successful, patients who failed RBBB correction showed a longer QRS duration (173.61 ± 23.63 ms vs. 148.06 ± 17.91 ms, *p* < 0.001) and a larger left ventricular size (67.90 ± 7.62 mm vs. 54.15 ± 7.67 mm, *p* < 0.001). Of the 10 patients, 4 had ischemic cardiomyopathy, and the scar limited the CSP delivery. Among the 10 patients, 4 patients accepted BiVP for the anticipated high proportion of ventricular pacing, and the other 6 patients accepted left ventricular septal pacing. However, no improvements were detected in LVEF values (33.3% ± 12.72% vs. 31.70% ± 9.98%, *p* = 0.337) after follow-up.

## Feasibility and safety of the CSP procedure

**FIGURE 1:**
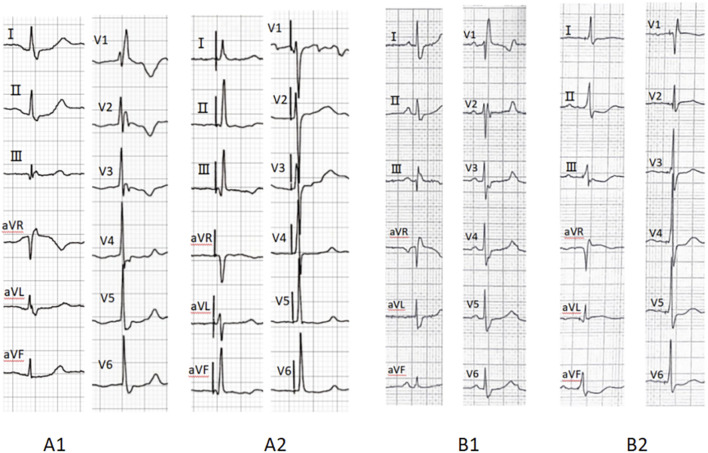
Preoperative ECG (A1) indicates RBBB, while A2 demonstrates complete correction of RBBB after His-bundle pacing. Preoperative ECG (B1) indicates RBBB, whereas B2 shows complete resolution of RBBB following left bundle branch pacing. RBBB, right bundle branch block; ECG, electrocardiogram.

Procedural outcomes are summarized in Table 2. Among the 78 patients with CSP, 13 patients underwent His-bundle pacing, while 65 underwent LBBP. The total procedural duration averaged 78.21 ± 22.93 min. The electrocardiograms before and after CSP are shown in Figure 1. The impedance significantly decreased compared to preoperative values (709.81 ± 203.18 Ω vs. 501.21 ± 128.49 Ω, *p* < 0.001). The R wave amplitude remained stable after follow-up (8.99 ± 5.77 mV vs. 10.96 ± 8.03 mV, *p* = 0.168). The HBP (1.25 ± 0.52 V vs. 1.24 ± 0.68 V, *p* = 0.987) and LBBP (1.08 ± 0.46 V vs. 1.04 ± 0.54 V, *p* = 0.618) thresholds remained stable during follow-up, and three patients exhibited increased thresholds. The heart rate significantly increased compared to preoperative values (48.23 ± 14.17 bpm vs. 65.23 ± 5.72 bpm, *p* < 0.001).

**TABLE 2:** Procedural parameters, electrocardiographic characteristics, and echocardiographic outcomes in patients with right bundle branch block at baseline and follow-up.

	Baseline (n = 78)	Follow-up (n = 78)	*p*-value
Procedural parameter			
R wave amplitude (mV)	8.99 ± 5.77	10.96 ± 8.03	0.168
Impedance (ohms)	709.81 ± 203.18	501.21 ± 128.49	<0.001
His capture threshold at 0.4 m (V)	1.25 ± 0.52	1.24 ± 0.68	0.987
LBBP threshold at 0.4 m (V)	1.08 ± 0.46	1.04 ± 0.54	0.618
Heart rate (bpm)	48.23 ± 14.17	65.23 ± 5.72	<0.001
QRS duration, ms	148.06 ± 17.91	121.87 ± 14.47	<0.001
Normal axis, n, %	39 (50.00%)	68 (87.18%)	<0.001
QRS duration in HBP, ms	154.92 ± 16.98	118.69 ± 14.30	<0.001
QRS duration in LBBP, ms	146.37 ± 17.69	121.95 ± 14.62	<0.001
NYHA class	2.97 ± 0.68	1.63 ± 1.08	0.001
LVEF (%)	43.79 ± 11.71	46.94 ± 10.06	0.020
LVEDD (mm)	54.15 ± 7.67	52.71 ± 7.67	0.016
LAD (mm)	44.72 ± 8.07	43.86 ± 8.42	0.114

HBP, His-bundle pacing; LBBP, left bundle branch pacing; LVEF, left ventricular ejection fraction; LVEDD, left ventricular end-diastolic diameter; LA, left atrium.

Among them, during the follow-up period, three patients had increased thresholds (defined as a ≥1 V increment), two in the HBP group and one in the LBBP group. No infections, thromboses, perforations, or lead dislodgements occurred during follow-up.

**FIGURE 2:**
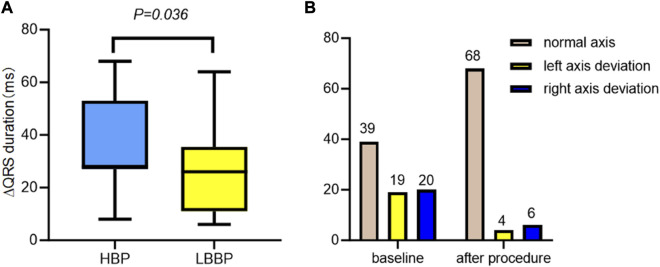
ECG characteristics with CSP, HBP, and LBBP in RBBB. **(A)** The change in the QRS duration in the HBP group was significantly shorter than that in the LBBP group. **(B)** The electric axis changes in CSP with RBBB patients in baseline and after procedure. ECG, electrocardiogram; CSP, conduction system pacing; HBP, His-bundle pacing; LBBP, left bundle branch pacing; RBBB, right bundle branch block.

ECG parameters are also detailed in Table 2. Of the 88 patients, 78 (88.6%) underwent successful CSP, with a pacing percentage of 78.21% ± 22.93%. Of these, 28 cases (35.89%) achieved complete RBBB correction. The QRS duration (148.06 ± 17.91 ms vs. 121.87 ± 14.47 ms, *p* < 0.001) reduced after RBBB correction. The normal electrical axis was more common (50.00% vs. 87.18%, *p* < 0.001) after RBBB correction. The paced QRS duration in patients with HBP was shorter than that in those with LBBP (121.95 ± 14.62 ms vs. 118.69 ± 14.30 ms, *p* = 0.036) (as shown in Figure 2).

### Cardiac performance and remodeling following CSP

**FIGURE 3:**
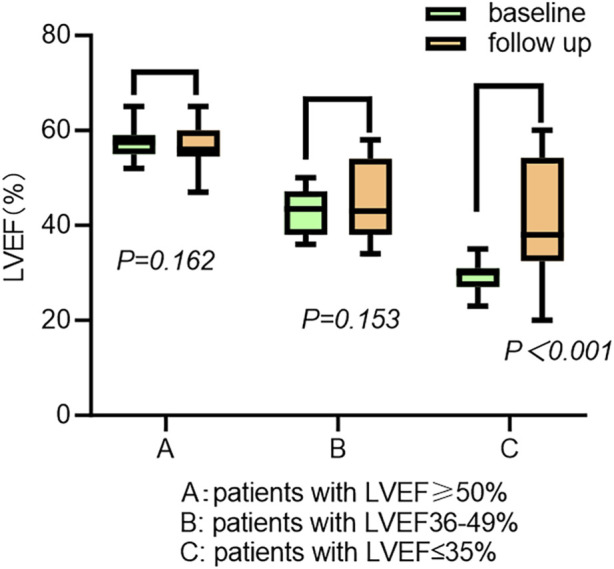
Changes in LVEF across LVEF levels before and after CSP.

The cardiac function gradually improved during the follow-up (Table 3). The New York Heart Association (NYHA) class (2.97 ± 0.68 vs. 1.63 ± 1.08, *p* < 0.001), LVEF (43.79% ± 11.71% vs. 46.94% ± 10.06%, *p* = 0.020), and left ventricular end-diastolic diameter (LVEDD) (54.15 ± 7.67 mm vs. 52.71 ± 7.67 mm, *p* = 0.016) showed significant improvements following CSP. The LAD was not enlarged in the patients (44.72 ± 8.07 mm vs. 43.86 ± 8.42 mm, *p* = 0.114) (Table 2). The subgroup analysis outcome is presented in Table 4. LVEF improved much more significantly in patients with LVEF < 50% (36.93% ± 7.67% vs. 43.60% ± 9.96%, *p* < 0.001). The LAD did not show significant enlargement in any patients (43.41 ± 5.53 mm vs. 42.98 ± 6.02 mm, *p* = 0.537). Further subgroup analysis demonstrated that LVEF improved (30.05% ± 2.76% vs. 41.42% ± 11.61%, *p* < 0.001) in patients with LVEF ≤35%more than in those with LVEF 36%–49% (43.18% ± 4.69% vs. 45.59% ± 7.95%, *p* = 0.153) and LVEF ≥ 50% (57.52% ± 2.69% vs. 54.81% ± 4.29%, *p* = 0.162) (as shown in Figure 3). The LVEDD decreased in patients with LVEF ≤35% (58.52 ± 7.83 mm vs. 53.52 ± 7.39 mm, *p* < 0.001) but not in those with LVEF 36%–49% (43.18% ± 4.69% vs. 45.59% ± 7.95%, *p* = 0.153).

**TABLE 3:** Changes in cardiac function during follow-up.

	Baseline	6 months	12 months	24 months	*p*-value
NYHA	2.97 ± 0.68	2.02 ± 0.46	1.73 ± 0.98	1.63 ± 1.08	0.008
Heart rate	48.23 ± 14.17	68.35 ± 6.87	64.34 ± 7.12	65.23 ± 5.72	0.010
LVEDD	54.15 ± 7.67	53.47 ± 12.01	52.52 ± 6.62	52.71 ± 7.67	0.034
LVEF	43.79 ± 11.71	44.27 ± 10.74	45.75 ± 10.46	46.94 ± 10.06	0.027
Pacing percentage		74.35 ± 26.87	72.34 ± 27.12	78.21 ± 22.9	0.473

NYHA, New York Heart Association; LVEF, left ventricular ejection fraction; LVEDD, left ventricular end-diastolic diameter.

**TABLE 4:** Echocardiographic outcomes and clinical outcome in patients with CSP.

	Baseline	Follow-up	*p*-value
LVEF <50% (n = 48)			
LVEF (%)	36.93 ± 7.67	43.60 ± 9.96	0.001
LVEDD (mm)	56.02 ± 7.56	54.59 ± 7.68	0.036
LAD (mm)	43.41 ± 5.53	42.98 ± 6.02	0.537
LVEF 36%–49% (n = 26)			
LVEF (%)	43.18 ± 4.69	45.59 ± 7.95	0.153
LVEDD (mm)	53.74 ± 6.67	51.91 ± 7.07	0.086
LAD (mm)	42.83 ± 5.51	41.61 ± 5.09	0.146
LVEF ≤35% (n = 22)			
LVEF (%)	30.05 ± 2.76	41.42 ± 11.61	0.001
LVEDD (mm)	58.52 ± 7.83	53.52 ± 7.39	0.014
LAD (mm)	44.05 ± 5.61	44.48 ± 6.70	0.713

LVEF, left ventricular ejection fraction; LVEDD, left ventricular end-diastolic diameter; LA, left atrium; CSP, conduction system pacing; HF, heart failure.

### Predictive factors for echocardiographic response

Among them, there were 48 patients with an LVEF<50%. Among these 48 patients, 27 achieved an LVEF improvement of ≥5% (27/48, 56.25%), and 17 (17/22, 77.27%) patients achieved improvement in ejection fraction among those with an LVEF ≤ 35%. The response ratio in patients with HBP and LBBP is not significantly different (62.50% vs. 55.00%, *p* = 0.696); however, the response ratio in patients with complete RBBB correction is higher than that in those with partial correction (70.59% vs. 45.16%, *p* = 0.037). Univariate and multivariate logistic regression analyses were used to identify predictors of echocardiographic response, as presented in Table 5. Our findings revealed that LVEF (OR = 0.112, 95% CI: 0.011–0.839, and *p* = 0.001) and ΔQRS (OR = 1.449, 95% CI: 1.292–2.445, and *p* = 0.021) were independent risk factors for predicting an echocardiographic response.

**TABLE 5:** Predictive factors for an echocardiographic response with RBBB patients and LVEF<50%.

	Univariate analysis			Multivariate analysis		
	OR	95% CI	*p*-value	OR	95% CI	*p*-value
Atrial fibrillation	0.468	0.136–1.611	0.228			
Ischemic cardiomyopathy	2.844	0.633–12.777	0.173			
HBP pacing modality	0.575	0.02–8.714	0.42			
Pacing percentage	0.124	0.914–1.011	0.961			
ΔQRS	1.165	1.058–1.283	0.002	1.449	1.292–2.445	0.021
ΔHR	1.105	1.033–1.197	0.041			
Baseline LVEF	0.279	0.131–0.593	0.001	0.112	0.011–0.839	0.001
ACEI/ARB/ARNI	1.100	0.332–3.640	0.876			
Beta-blocker	1.212	0.413–1.514	0.155			
MRA	1.132	0.494–2.530	0.586			
SGLT2i	1.167	0.617–2.744	0.480			

HBP, His-bundle pacing; LBBP, left bundle branch pacing; LVEF, left ventricular ejection fraction; ΔQRS, changes in QRS duration; ΔHR, alterations in heart rate; ACEI, angiotensin-converting enzyme inhibitor; ARB, angiotensin receptor blocker; ARNI, angiotensin receptor–neprilysin inhibitor; MRA, mineralocorticoid receptor antagonist; SGLT2i, sodium–glucose cotransporter 2 inhibitor.

## Discussion

### Main findings

The study evaluated CSP in patients with RBBB and HF and presented several key findings: CSP was associated with significant improvements in cardiac function among patients with RBBB and HF, particularly those with RBBB and LVEF ≤ 35%. LVEF and ΔQRS were independent predictors of the echocardiographic response. CSP notably shortened the QRS duration and promoted RBBB reversal, particularly in patients with HBP, thereby improving physiological ventricular activation. The pacing parameters remained stable over a 2-year follow-up, confirming long-term safety and feasibility.

### Electrophysiological characteristics of RBBB reversal following CSP

Previous studies have indicated that CSP is a viable option for RBBB reversal (Zhu et al., 2021; Ponnusamy et al., 2025). This present study further demonstrates that CSP significantly reduces QRS duration (148.06 ± 17.91 ms vs. 121.87 ± 14.47 ms, *p* < 0.001) following CSP and substantially enhances electrical axis normalization compared to baseline (50.00% vs. 87.18%, *p* < 0.001).

**FIGURE 4:**
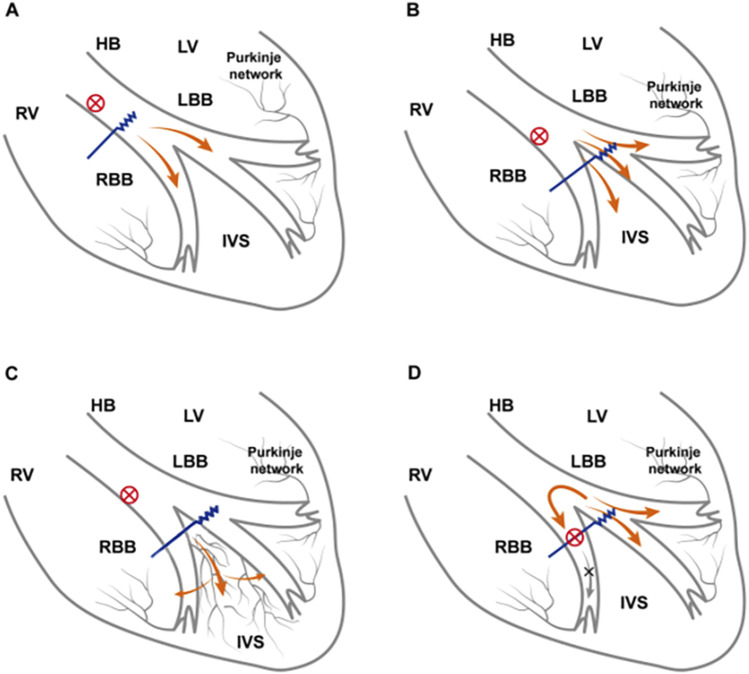
Electrophysiological mechanism of CSP in RBBB reverse. **(A)** RBBB is located within the His bundle. HBP is performed distal to the His bundle and across the lesion, correcting RBBB or relying on high-voltage pacing output to capture RBBB. **(B)** As the pacing output voltage increases, the left bundle branch and the local adjacent septal myocardium are captured. Greater capture of the septal myocardium partially compensates for the right ventricle excitation delay caused by RBBB and may even capture the right bundle branch beyond the conduction block, thereby correcting RBBB. **(C)** LBBP lead tips directly capture electrical activity of the septal Purkinje network, which forms interconnections with both left and right bundle branches, enabling rapid impulse propagation through the His–Purkinje system. **(D)** The block site is located at the distal right bundle branch and thus cannot be corrected by LBBP through any approach. CSP, conduction system pacing; LVEF, left ventricular ejection fraction; RBBB, right bundle branch block; HBP, His-bundle pacing; LBBP, left bundle branch pacing.

The mechanisms underlying paced QRS modulation are as follows: (A) if the block is located in the His bundle branch, according to the longitudinal dissociation theory, distal HBP can bypass the block to correct RBBB; alternatively, high pacing output can also capture the right bundle branch if the lead is located near the block site (Mahmud and Jamal, 2021). (B) If the block is located beyond the His bundle branch, the reversal mechanisms are as follows: both the left bundle branch and the adjacent local septal myocardium are activated, and the septal myocardium partially offsets the right ventricular activation delay (Ponnusamy et al., 2024; Li et al., 2019; Li et al., 2020). Anode capture further partially compensates for the right ventricular electrical delay (Huang et al., 2019). (C) An intramyocardial Purkinje network has been identified within the ventricular septum, which might provide a unifying explanation for normalization bundle branch conduction following LBBP (Liu et al., 2025). All the mechanisms are shown in Figure 4. However, CSP cannot reverse the distal right bundle branch block.

The total reversal rate is 88.81%, including 13 patients with HBP and 65 patients with LBBP. Among the 10 patients who failed reversal, QRS duration was as long as 170 ms, suggesting that a longer QRS duration may be associated with the failure of RBBB reversal. However, for distal RBBB, CSP failed to reverse conduction impairment, regardless of pacing output, due to the distal block location (Wu et al., 2020).

Complete RBBB results in electrical asynchrony between the left and right ventricles and within the right ventricle. The ECG changes induced by LBBP differ from intrinsic RBBB, and right ventricular activation remains synchronous, despite interventricular asynchrony following LBBP (Ozpak et al., 2022). Partial resolution of RBBB may result from right ventricular activation patterns. Over time or at lower pacing voltages, passive pre-excitation of the right ventricle (from the septum to the free wall) may lead to partial correction, which may explain the incomplete normalization after CSP (Mahmud and Jamal, 2021).

### Cardiac performances following CSP in different LVEF categories

Current data demonstrated that CRT is not associated with reduced mortality or heart failure hospitalizations in patients with non-LBBB QRS morphology. However, the role of CRT in patients with wide QRS and non-LBBB morphology remains clinically uncertain (Cunnington et al., 2015). Some studies showed that patients with RBBB and concomitant left anterior fascicular block or left posterior fascicular block may also potentially benefit from CRT as either anterior or posterior block can induce partially delayed left ventricular activation (Dennis et al., 2022; Naruse et al., 2014). Additionally, there may be significant benefits from CRT in patients with HF and RBBB who also have coexisting AV block (Vijayaraman et al., 2022).

A previous study has established that among patients with an LVEF <35%, those with RBBB exhibited significantly more myocardial scarring than those with LBBB (Strauss et al., 2013). The increased scarring was associated with more pronounced right ventricular dysfunction and more severe congestive symptoms (Pellicori et al., 2015). Consequently, the use of CSP, which can significantly reduce QRS duration, may potentially improve clinical outcomes. Our study demonstrated that CSP improved cardiac function in patients with RBBB and an LVEF ≤35%. This finding demonstrated the benefits of CSP in this patient population, likely due to its role in restoring electrical synchronization.

Sharma et al. (2018a) demonstrated that HBP was associated with a significant improvement in left ventricular function in patients with RBBB and reduced LVEF. Similarly, Vijayaraman et al. (2022) also found that LBBP was a feasible alternative for patients with RBBB and HF. In this present study, it was further revealed that patients with LVEF ≤35% exhibited significant LVEF improvement following CSP.

## Limitations

This study was a retrospective cohort study conducted at a single center. Larger, randomized, multicenter studies may be necessary to validate these findings. The outcome comparison between HBP and LBBP was not performed due to the limited sample size. Owing to the lack of direct comparison with right ventricular pacing and the absence of a dedicated control group, further validation of our findings through large-scale, prospective, randomized controlled trials is warranted. Additionally, the presence of numerous confounding factors, such as intraventricular conduction block, first-degree atrioventricular block, and concomitant underlying diseases, may all potentially affect the study results.

## Conclusion

CSP is safe and effective for patients with RBBB and HF, with particularly notable improvements in cardiac performance among those with severely reduced LVEF.

## Data Availability

The original contributions presented in the study are included in the article/Supplementary Material; further inquiries can be directed to the corresponding author.

## Glossary

CSP: conduction system pacing
RBBB: right bundle branch block
HF: heart failure
HBP: His-bundle pacing
LBBP: left bundle branch pacing
LVEF: left ventricular ejection fraction
LVEDD: left ventricular end-diastolic diameter
LAD: left atrium diameter
NYHA: New York Heart Association
LBBB: left bundle branch block
CRT: cardiac resynchronization therapy
BiVP: biventricular pacing
HFrEF: heart failure with reduced left ventricular ejection fraction
LVAT: Stim-left ventricular active time
LV: left ventricular
AV: atrioventricular
HFpEF: heart failure with preserved left ventricular ejection fraction
ECG: electrocardiogram
ACEI: angiotensin-converting enzyme inhibitor
ARB: angiotensin receptor blocker
ARNI: angiotensin receptor–neprilysin inhibitor
MRA: mineralocorticoid receptor antagonist
SGLT2i: sodium–glucose cotransporter 2 inhibitor
ΔQRS: changes in QRS duration
ΔHR: alterations in heart rate
AF: atrial fibrillation
BMI: body mass index
CR: creatinine
BNP: B-type natriuretic peptide
QRSd: QRS duration
CI: confidence interval
OR: odds ratio
