# Supplementary material for: Is conduction system pacing improving cardiac performance in patients with right bundle branch block and heart failure?
Source: Front Physiol. 2025 Nov 25;16:1690243. doi: 10.3389/fphys.2025.1690243 (PMC12685662; doi:10.3389/fphys.2025.1690243)
Supplement: Supplementary file 1 [file Table1.doc]

**Supplementary materials**

Based on our data review, we conducted a retrospective analysis of 17 patients with normal LVEF who received right ventricular septal pacing during the same period and had echocardiographic follow-up data.

Among the 17 patients, the mean age stood at 66.59 ± 10.51 years, with males constituting 58.82% of the cohort, and atrial fibrillation observed in 70.59% of the cases.

During the same study period, totally 17 patients with RBBB who received right ventricular septal pacing were enrolled. After a follow-up of 19.44 ± 10.59 months, these patients showed a significant reduction in LVEF (57.43 ± 4.00% vs. 53.62 ± 6.83%, *P* = 0.025) and a marked increase in QRS duration (140.49 ± 16.15 ms vs. 163.19 ± 12.55 ms, *P* = 0.001).

Supplementary Table 1. Baseline Characteristics of enrolled patients

| Characteristic |  |
| --- | --- |
| Male | 10（58.82%） |
| Age（y） | 66.59±10.51 |
| BMI（Kg/m^2^） | 24.80±3.25 |
| Hypertension | 10（58.82%） |
| Diabetes mellitus | 4（23.50%) |
| atrial fibrillation | 12（70.59%） |
| QRSd (ms) | 140.49±16.15 |
| Follow up duration (months) | 19.44 ± 10.59 |

BMI, body mass index; CR, creatinine; BNP, B-type natriuretic peptide; NYHA, New York Heart Association; QRSd, QRS duration;

Supplementary Table 2. Changes in clinical outcomes before and after surgery in the right ventricular septal pacing group（Supplementary materials 1）

|  | baseline | follow up | *P* value |
| --- | --- | --- | --- |
| QRSd (ms) | 140.49±16.15 | 163.19±12.55 | 0.001 |
| LAD | 44.22±6.42 | 45.64±5.87 | 0.128 |
| LVEDD | 48.78±6.42 | 49.74±4.89 | 0.079 |
| LVEF | 57.43 ± 4.00 | 53.62 ± 6.83 | 0.025 |

QRSd, QRS duration; LVEF, left ventricular ejection fraction;LVEDD, left ventricular end-diastolic diameter; LAD, left atrium diameter
